# An Integrated Strategy of Chemical Fingerprint and Network Pharmacology for the Discovery of Efficacy-Related Q-Markers of *Pheretima*

**DOI:** 10.1155/2022/8774913

**Published:** 2022-10-04

**Authors:** Ye Shang, Suyi Liu, Chunxiao Liang, Kunze Du, Shujing Chen, Jin Li, Hua Jin, Yanxu Chang

**Affiliations:** ^1^State Key Laboratory of Component-based Chinese Medicine, Tianjin University of Traditional Chinese Medicine, Tianjin 301617, China; ^2^Tianjin Key Laboratory of Phytochemistry and Pharmaceutical Analysis, Tianjin University of Traditional Chinese Medicine, Tianjin 301617, China; ^3^College of Traditional Chinese Medicine, Tianjin University of Traditional Chinese Medicine, Tianjin 300193, China

## Abstract

*Pheretima,* one of the animal-derived traditional Chinese medicines, has been wildly used in various cardiovascular and cerebrovascular diseases, including stroke, coronary heart disease, hyperlipidemia, and hyperglycemia. However, it was still a big challenge to select the quality markers for *Pheretima* quality control. The fingerprint and network pharmacology-based strategy was proposed to screen the efficiency related quality markers (Q-Markers) of *Pheretima*. The ratio of sample to liquid, ultrasonic-extraction time, temperature, and power were optimized by orthogonal design, respectively. The chemical fingerprint of forty batches of *Pheretima* was established, and six common peaks were screened. The network pharmacology was used to construct the *Pheretima*-Components-Targets-Pathways-Stroke network. It was found that six potential efficacy Q-markers in *Pheretima* could exert the relaxing meridians effect to treat stroke through acting on multiple targets and regulating various pathways. A simple HPLC-DAD method was developed and validated to determine the efficacy Q-markers. Grey relational analysis was used to further verify the relation of potential efficiency related quality markers with the anticoagulation activity of *Pheretima*, which indicated that the contents of these markers exhibited high relationship with the anticoagulation activity. It was concluded that hypoxanthine, uridine, phenylalanine, inosine, guanosine, and tryptophan were selected as quality markers related to relaxing meridians to evaluate the quality of *Pheretima*. The fingerprint and network pharmacology-based strategy was proved to be a powerful strategy for the discovery of efficiency related Q-markers of *Pheretima.*

## 1. Introduction

Stroke remained the foremost cause of disability and the third leading cause of mortality all over the world. The demand for stroke prevention and recovery services was growing [1]. Stroke was usually caused by various risk factors. One was direct factors, including blockage or bleeding of the anterior cerebral artery or cerebral artery, and the other was indirect causes such as smoking, drug abuse, hypertension, obesity, and so on [2, 3]. Stroke could induce cerebral ischemia. Thereafter, the vascular endothelial cells release generous inflammatory factors and reactive oxygen species (ROS). Then neurocyte damage would occur rapidly and result in facioplegia and paralysis [4, 5]. Aspirin and clopidogrel were often used to treat stroke [6, 7]. However, long-term oral administration might cause gastrointestinal discomfort, dizziness, nausea, and many other side effects [8, 9]. Compared with chemical medicines, traditional Chinese medicines (TCMs) have been broadly used in many countries for centuries due to the significant therapeutic effect and fewer side effects. There were many types of TCMs used for stroke treatment, such as *Pheretima, Chuanxiong rhizome, Rhei radix et rhizome,* and so on [10].

*Pheretima*, also named Dilong, was the dried body of *Pheretima aspergillumPheretima vulgaris* Chen, *Pheretima guillelmi, and Pheretima pectinifera* Michaelsen [11]. *Pheretima* was an animal-derived TCM. It was firstly recorded in *Sheng Nong's Herbal Classic* and listed as “low grade.” *Pheretima* has been widely used for treating high fever, arthralgia, lung heat, cough, and asthma, etc. [12]. Because of the notable dredging collaterals efficacy of *Pheretima*, it has been used for many prescription compatibilities such as Buyang Huanwu decoction [13], Naoxintong capsule [14], Shentong Zhuyu decoction [15], Qiyu Sanlong decoction [16], and so on. There were a number of researches indicated that the ingredients of *Pheretima* mainly included amino acids, nucleobases, nucleosides, fatty acids, polypeptides, and lumbrokinase [17–21]. These ingredients contributed diverse pharmacological activities including anti-inflammatory, antitumor, antifibrosis, and nerve protecting effects [22–25]. However, the efficacy-dependent chemical markers of *Pheretima*quality control were ambiguous because of the complexity of composition.

The screening of TCMs quality markers (Q-markers) has attracted extensive attention from scholars all over the world. The concept of Q-markers focused on quality transitivity, specificity, differences, and dynamic changes of the ingredients with the core of component efficacy [26]. Nowadays, many experts devoted themselves to screening Q-marker of TCMs with different methods. For instance, a ultraperformance liquid chromatography tandem mass spectrometry (UPLC-MS/MS) based pharmacokinetics-pharmacodynamics research was carried out to confirm wilforine served as a potential Q-marker of Tripterygium glycosides tablet [27]. For *Typhae Pollen*, ultraperformance liquid chromatography and tandem quadrupole time of flight mass spectrometry (UPLC-Q-TOF-MS) together with evaluation of thrombin activity was used to discovery bioactive Q-markers [28].

Chromatography fingerprint played a key role in TCMs quality control. Fingerprints were constructed by liquid chromatography with ultraviolet detector (LC-UV), liquid chromatography tandem mass spectrometry (LC-MS), gas chromatography (GC), nuclear magnetic resonance spectroscopy (NMR), thin-layer chromatography (TLC), etc. [29–31]. Unsupervised data analysis was an important methodology for data treatment of TCMs. It mainly includes correlation coefficient, congruence coefficient, Euclidean distance, and so on. This statistical analysis could identify common peaks and calculate similarity values between two complex TCM samples [32]. Network pharmacology was considered as an appropriate technology of traditional Chinese medical pharmacotherapy that emphasized the concept of multitarget and multicomponent therapeutics. Network pharmacology was usually proposed as a desirable tool to uncover the potential targets and mechanisms of TCMs. It has been successfully applied to various TCMs and formulas, such as *potentilla Discoloris Herba* [33], *Cistanches Herba* [34], and *Huashi Baidu formula* [35].

In the present study, the chemical fingerprint of TCM and network pharmacology-based strategy was proposed to screen the efficiency-related quality markers (Q-Markers) of *Pheretima*. The anticoagulation activity was used to verify the relation of Q-Markers with efficacy. This strategy was successfully employed to screen efficacy-related Q-markers for improving quality control and providing clinical reference for *Pheretima*.

## 2. Materials and Methods

### 2.1. Chemicals and Reagent

Hypoxanthine, uridine, adenine, inosine, guanosine, and adenosine were purchased from Chengdu Desite Bio-Technology Co., Ltd. (Chengdu, China). Phenylalanine and tryptophan were bought from Shanghai Aladdin Biochemical Technology Co., Ltd (Shanghai, China). Potassium dihydrogen phosphate (KH_2_PO_4_) was purchased from Tianjin Fengchuan Chemical Reagent Co., Ltd. Thrombin, fibrinogen, and phosphate buffer saline (PBS, pH 7.2–7.4, 0.01 M) were purchased from Beijing Solarbio Science and Technology Co., Ltd. (Beijing, China). HPLC-grade methanol (MeOH) was obtained from Fisher (Leicestershire, UK). Ultrapure water was obtained from a Milli-Q academic ultrapure water system (Millipore, Milford, MA, USA).

Forty batches of *Pheretima*samples were purchased from medicine markets and pharmacies in different provinces in China. The *Pheretima* samples were dried at 60°C under reduced pressure before being pulverized into homogeneous powder (through a 65-mesh sieve).

Although the detailed information on *Pheretima* species was not identified because they have been processed into decoction pieces. The samples were authenticated as *Pheretima* according to *Chinese Pharmacopeia* by Prof. Yanxu Chang (Tianjin University of Traditional Chinese Medicine).

### 2.2. Preparation of Standard Stock Solutions, Thrombin Solution, and Fibrinogen Solution

Eight reference substances were accurately weighed and dissolved with 0.05 M sodium hydroxide (hypoxanthine at the concentration of 1 mg/mL), 0.05 M hydrochloric acid (adenine and guanosine at the concentration of 1 mg/mL), and water (uridine, tryptophan, and adenosine at the concentration of 1 mg/mL, inosine and phenylalanine at the concentration of 2 mg/mL), respectively. The standard stock solutions were mixed and diluted to different concentrations by water to establish standard curves. All standard stock solutions were stored at 4°C before being used.

Thrombin and fibrinogen were dissolved in normal saline (1000 U/mL) and PBS (5 mg/mL), respectively. The thrombin solution was stepwise diluted to 40 U/mL by normal saline. All thrombin and fibrinogen solutions were stored in ice bath before being used.

### 2.3. HPLC-DAD Analysis

Thermo Scientific Ultimate 3000 HPLC system consisting of a ternary pump, autosampler, thermostated column compartment, and diode array detector (DAD) was employed throughout the analysis. All separation was performed on a Welch AQ-C18 HPLC column (4.6 mm × 250 mm, 5 *μ*m). The flow rate was set at 0.8 mL/min, and the column temperature was fixed at 35°C. The mobile phase was composed of 10 mM H_2_KPO_4_ aqueous solution (*A*) and methanol (B) with the gradient elution: 0∼20 min, 1% B; 26∼30 min, 3∼5% B; 40∼45 min, 7∼10% B, 60∼63 min, 10∼1% B and the reequilibration time of elution was 8 min. The wavelength of the detector was set at 210 nm.

### 2.4. Optimization of Ultrasonic-Assisted Extraction

Single-factor experiments were firstly employed to define the rang of each specific parameter in the ultrasonic-assisted extraction. *Pheretima* powder (20 mg/mL) was initially used as the object for the optimization of related conditions. Four single-factor experiments were sequential implemented and repeated three times. The ratio of sample to liquid, extraction temperature, power, and time was optimized, respectively. The relevant parameters of the extraction program were initially established and employed in the following study.

Considering the interaction of different factors, a four-factor and three-level (*L*_9_3^4^) orthogonal design was proposed correspondingly for ultrasonic-assisted extraction. Taking total content of analytes as index, a ratio of sample to solvent (A, mg/mL), extraction time (B, min), temperature (C, °C) and power (*D*, W) was tested in the orthogonal design. Based on the water as the extraction solvent, the experiments were performed. The experimental scheme and orthogonal header design are shown in Table 1.

### 2.5. Establishment of *Pheretima* HPLC-DAD Fingerprint 

The optimized method was used to extract forty batches of *Pheretima* samples. As a result, 100 mg samples were weighted into 10 mL volumetric flask and then soaked 10 min after adding water to the scale mark. Ultrasonic-assisted extraction (40 kHz, 220W) was performed in a water bath at 40°C for 40 min. Each extract was centrifuged at 14000 rpm for 10 min and through a 0.22 *μ*m membrane. The subsequent filtrates were injected into the HPLC-DAD system to establish *Pheretima* fingerprint. The recognized common peaks were used as potential active components for the following study.

### 2.6. Establishment of P*heretima*-Components-Targets-Pathways-Stroke Network

#### 2.6.1. Target Fishing

The targets of potential active components were obtained from Pharm Mapper database (http://www.lilab-ecust.cn/pharmmapper/). Stroke related targets were obtained from the OMIM database (https://omim.org/), DrugBank database (https://go.drugbank.com/), and GeneCards database (https://www.genecards.org/).

#### 2.6.2. Establishment of Protein-Protein Interaction (PPI)

The common targets between potential active components and stroke were imported into STRING (Version 11.5, https://string-db.org/) and Cytoscape software (version 3.7.2) for visualization and statistical analysis.

#### 2.6.3. Gene Ontology (GO) and Kyoto Encyclopedia of Genes and Genomes (KEGG) Enrichment Analysis

For preliminarily exploring the relevant mechanism of key nodes, GO, and KEGG enrichment analyses were performed to gather the corresponding biological functions and processes via the DAVID Bioinformatics Resources (version 6.8, https://david.ncifcrf.gov/summary.jsp). GO enrichment analysis was mainly involved in GO biological process (BP), GO molecular function (MF), and GO cellular component (CC). The result of KEGGen richment was plotted by WeiShengxin, an online platform for visualization (http://www.bioinformatics.com.cn/).

#### 2.6.4. Establishment of *Pheretima*-Components-Targets-Pathways-Stroke Network

All related targets and pathways were summarized, and the network was constructed using Cytoscape software (version 3.7.2).

### 2.7. Anticoagulation Activity Assay

The thrombin titration method reported in *Chinese Pharmacopeia* was used to evaluate the anticoagulation activity of *Pheretima* [11, 36, 37]. Briefly, 200 *μ*L fibrinogen (5 mg/mL in PBS) and 100 *μ*L *Pheretima* extract (diluted or concentrated from 200 mg/mL) were added to 2 mL centrifuge tube. They were shaken gently and incubated for 5 min at 37°C. Thereafter, 5 microliters of thrombin (40 U/mL in normal saline) were added per minute until fibrous coagulation occurred. The consumed volume of thrombin was used to evaluate the anticoagulation activity of *Pheretima*. The samples were grouped according to anticoagulation activity by SPSS software (version 25). The formula of anticoagulation activity was as follows:

(1) $$\begin{array}{c} U=\frac{C1V1}{C2V2}. \end{array}$$

U (U/g) represented the active unit of thrombin per Gram. C_1_ (U/mL) and V_1_ (*μ*L), respectively, expressed the concentration and consumed volume of thrombin. C_2_ (g/mL) and V_2_(*μ*L) showed the concentration and dosage of samples.

## 3. Results and Discussion

### 3.1. Optimization of Chromatographic Conditions

To obtain good chromatographic separation, the HPLC conditions include a concentration of additive (0, 5, and 10 mM KH_2_PO_4_), a flow rate of mobile phase (0.6, 0.8, and 1.0 mL/min), and detective wavelength (210, 254, and 280 nm) were comprehensively investigated. Considering the peak resolutions, shapes and responses, the best separation was achieved when 10 mM H_2_KPO_4_ was selected as an additive to the water phase. The flow rate and detective wavelengths were 0.8 mL/min and 210 nm, respectively.

### 3.2. Optimization of Sample Extraction

The optimized extraction program was obtained by the orthogonal experimental design. Although the optimized level of extraction time and extraction temperature was 60 min and 60°C, respectively (Table 1). There was no significant difference between each level. In order to protect the environment and save time and energy, 100 mg samples were weighted into 10 mL volumetric flask and soaked 10 min after water was added to the scale mark (Table 1). Ultrasonic-assisted extraction (40 kHz, 220 W) was performed in a water bath at 40°C for 40 min.

### 3.3. The Chemical Fingerprint of Forty Batches of P*heretima*

**Figure 1:**
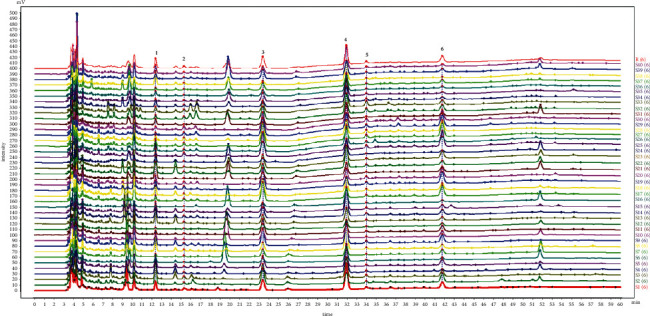
The chemical fingerprint of 40 batches of *Pheretima*. (1) Hypoxanthine, (2) Uridine, (3) Phenylalanine, (4) Inosine, (5) Guanosine, and (6) Tryptophan.

The optimized chromatographic conditions and extraction method were successfully applied to construct the chemical fingerprint of *Pheretima* by HPLC-DAD method (Figure 1). The Similarity Evaluation System (version 2012) was a software that was able to generate a control fingerprint and calculate the similarity value between each sample with the control fingerprint. The control fingerprint of *Pheretima* was created by the median method. Six common peaks were identified as hypoxanthine, uridine, phenylalanine, inosine, guanosine, and tryptophan by comparing with the retention time and extracted DAD spectra of reference standards. Finally, the similarity value of each sample with a control fingerprint was calculated. It was found that the similarity values of most samples were larger than 0.90, which indicated the qualities of most samples are similar. These qualities were various with the other samples, including S2, S17, S21, S25, S27, and S38 (Table S1).

### 3.4. Network Pharmacology Analysis

#### 3.4.1. Analysis of PPI Network

**Figure 2:**
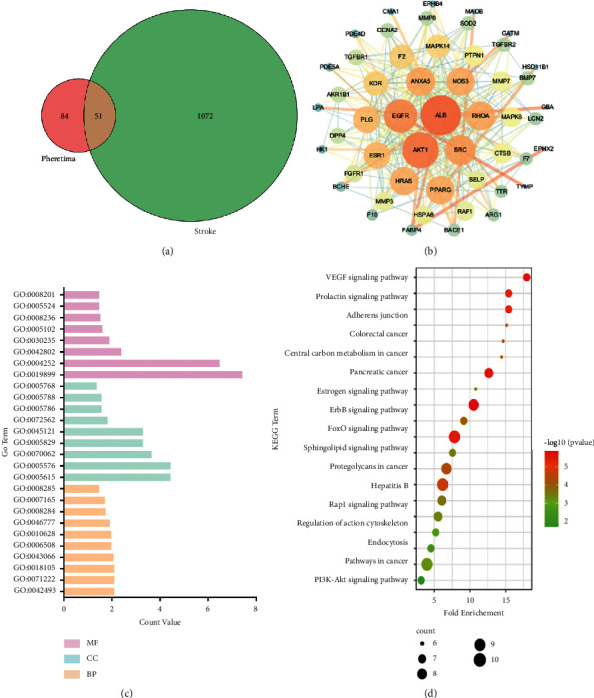
The Venn of six components and stroke targets (a). The PPI network of 51 targets (The size and color represented the degree value. Larger and redder meant a greater degree value and betweenness of nodes and edges) (b). The items of GO enrichment (c). The items of KEGG pathways enrichment (d).

There were 135 related targets of potential active components by PharmMapper database with Norm Fit ≥0.6. Among them, 51 targets were the same as disease targets (Figure 2(a)). To reveal the function of 51 targets against stroke, a PPI network was successfully visualized by the STRING database and analyzed by Cytoscape software. The network contained 51 nodes and 297 edges.

In view of the fact that node degree value could not only reflect the number of nodes connected to it, but also incarnated its importance. As shown in Figure2(b), the biggest node was ALB, which was serum albumin that can regulate the colloidal osmotic pressure of blood. ALB was a valuable diagnostic marker of many diseases, such as rheumatoid arthritis, ischemia, and diseases that need monitoring of glycemic control. Clinically, ALB has been extensively used for disease treatment, including shock, hemorrhage, and hypovolemia [38]. AKT1 was one of three closely related serine/threonine-protein kinases (AKT1, AKT2, and AKT3). It played an important role in diverse biological progress, for instance, proliferation, migration, cell growth, angiogenesis, and vasorelaxation [39]. NOS3 was the most crucial isoform of nitric oxide synthase in endothelial cells. Nitric oxide synthase (NOS) could produce nitric oxide (NO), which was an important vasodilator with a well-established role in cardiovascular homeostasis [40]. It has been confirmed that NOS-knocked mice had more severe brain damage after cerebral stroke. The mechanism might be that NO can protect brain tissue by regulating hemodynamics [41].

#### 3.4.2. GO Enrichment Analysis

After filtering by both *P* value and FDR (false discovery rate) less than 0.05, GO enrichment has enriched 45, 9, and 8 terms in BP, MF, and CC, respectively (Figure2(c) and Table S2). According to the rank of “count” value, BP terms were mainly related to response to the drug (GO:0042493), cellular response to lipopolysaccharide (GO:0071222), peptidyl-serine phosphorylation (GO:0018105), negative regulation of apoptotic process (GO:0043066), proteolysis (GO:0006508), positive regulation of gene expression (GO:0010628), protein autophosphorylation (GO:0046777), positive regulation of cell proliferation (GO:0008284), signal transduction (GO:0007165) and negative regulation of cell proliferation (GO:0008285). In the aspect of MF, there were enzyme binding (GO:0019899), serine-type endopeptidase activity (GO:0004252), identical protein binding (GO:0042802), nitric oxide synthase regulator activity (GO:0030235), receptor binding (GO:0030235), serine-type peptidase activity (GO:0008236), ATP binding (GO:0005524), and heparin binding (GO:0008201). CC was related to extracellular space (GO:0005615), extracellular region (GO:0005576), extracellular exosome (GO:0070062), cytosol (GO:0005829), membrane raft (GO:0045121), blood microparticle (GO:0045121), plasma membrane (GO:0005886), endoplasmic reticulum lumen (GO:0005788), and endosome (GO:0005768).

#### 3.4.3. KEGG Analysis

Finally, a total of 63 KEGG pathways were obtained. In addition, after screening the pathways with FDR less than 0.05 and ranking based on the “count” value, the top-20 terms were selected for visualization (Figure 2(d)). The result indicated that the potential pathways of *Pheretima* against stroke, including VEGF signaling pathway (hsa04370), Rap1 signaling pathway (hsa04015), MAPK signaling pathway (hsa04010), PI3K-Akt signaling pathway (hsa04151), adherens junction (hsa04520), FOXO signaling pathway (hsa04068), and so on.

As numerous experimental data showed, inflammatory injury and oxidative stress were the pivotal pathological reaction of stroke. VEGF (vascular endothelial growth factors) signaling pathway was a crucial reducer in both physiologic and pathologic angiogenesis. It possessed a combined mechanism that relieved the injury on brain tissue after ischemia to regulate angiogenesis, neuroprotection, and the migration of neuronal stem cells into the ischemic area. It was noteworthy that the PI3K-Akt pathway was also enriched. PI3K-Akt signaling pathway was one of the downstream pathways of the VEGF signaling pathway. VEGF signaling pathway could promote phosphorylation of Akt, which was able to activate nitric oxide synthase (NOS). The function of NOS has already been mentioned above [42]. MAPK (mitogen-activated protein kinase) signaling pathway regulated a wide range of biological progress, such as cell growth, inflammation, and stress responses. It has been reported that *Pheretima* extracts could inhibit the MAPK signaling pathway through ERK_1/2_ and p38 [43].

#### 3.4.4. *Pheretima*-Components-Targets-Pathways-Stroke Network Analysis

**Figure 3:**
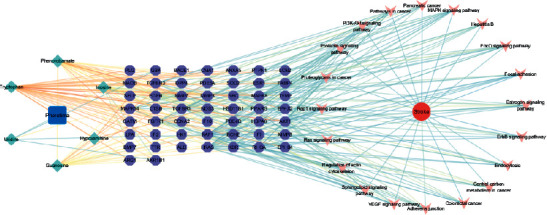
The *Pheretima*-Components-Targets-Pathways-Stroke Network. Square, rhombus, hexagon, vee, and circle represent *Pheretima*, components, targets, pathways, and stroke, respectively.

A*Pheretima*-Components-Targets-Pathways-Stroke network has been constructed (Figure 3). It was composed of 79 nodes (containing 6 compounds, 51 targets and 20 pathways) and 281 edges. According to the network, six components of *Pheretima* could exert the relaxing meridians effect to treat stroke through acting on multiple targets and regulating various pathways.

Atherosclerosis was one of the risk triggers of stroke, and it was fatal for stroke patients as it induced hypertension and venous thrombosis. High-level of hypoxanthine and xanthine in hyperlipidemia patients were used to maintain fluid circulation homeostasis. It has been confirmed that organisms would act on the immune system and upgrade the concentrations of hypoxanthine and xanthine when a disturbance of blood circulation occurs [44, 45]. Furthermore, purine nucleosides, such as hypoxanthine, xanthine, and so on, have been considered as diagnostic biomarkers of tissue ischemia [46]. *In vivo*, tryptophan could be metabolized to several kynurenines with antioxidant activity. It has been reported that kynurenines could scavenge reactive oxygen species (ROS) and ease oxidative stress in ischemic tissue. Kynurenic acid, one of the kynurenines, was often considered a neuroprotection agent in reducing brain damage [47]. Overall, a total of six compounds were selected as efficacy-related Q-markers of *Pheretima*. Then, these six efficacy-related Q-markers of *Pheretima* were simultaneously determined by HPLC-DAD according to the chromatography conditions of a fingerprint.

### 3.5. Method Validation

#### 3.5.1. Linearity and Sensitivity

The standard curves of all analytes were contracted by plotting the peak area against concentration. Their correlation coefficients (*r*^2^) were more than 0.9994. The limit of detection (LOD) and limit of quantitation (LOQ) of analytes correspond to the concentration level at signal-to-noise ratios (S/N around 3 and 10), respectively. The LOD and LOQ ranged from 0.04 to 0.44 *μ*g/mL and 0.13 to 1.21 *μ*g/mL, respectively (Table 2).

#### 3.5.2. Precision, Stability, and Repeatability

The precision assay was expressed as relative standard deviation (RSD) by detecting eight compounds in *Pheretima* solution with six replicates. The stability was assessed with RSD by repeating the determination six times. *Pheretima* solution was deposited in an autosampler and analyzed at 0, 2, 4, 8, 12, and 24 h. The repeatability could be obtained by determining the target ingredients of six replicates using the configured system. As the results (Table 3), the RSDs of precision, stability, and repeatability were in the range of 0.36% to 2.85%, 1.35% to 4.35%, and 1.21% to 4.95%, respectively.

#### 3.5.3. Accuracy

The accuracy was assessed by recoveries of spiking samples. A 50 mg aliquot of *Pheretima* was extracted by pooling known amounts of standard solutions with the well-defined method. The formula of recovery was presented as follows:

(2) $$\begin{array}{c} \text{recovery}\left(\%\right)=\frac{\text{spiked sample amount}-\text{sample amount}}{\text{adding amount}}\times 100\%. \end{array}$$

The range of eight components recoveries was from 98.1% to 102%, and RSD ranged from 1.97% to 3.28%. The detailed data is displayed in Table 3.

### 3.6. Quantitative Analysis

**Figure 4:**
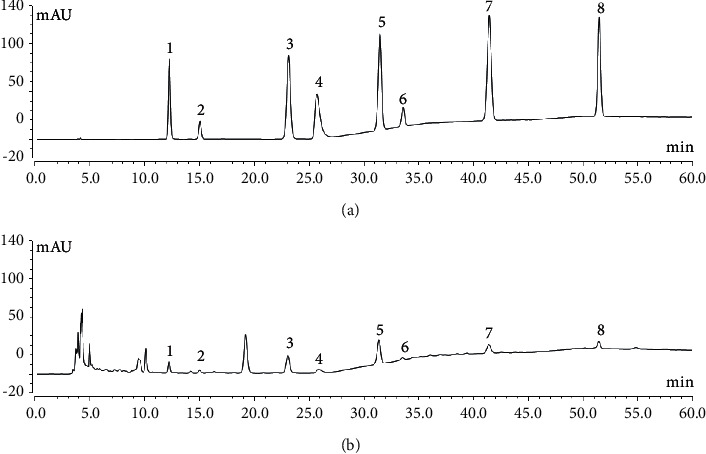
Representative chromatograms of mixed reference standards (a) and *Pheretima* (b). 1. Hypoxanthine, 2. Uridine, 3. Phenylalanine, 4. Adenine, 5. Inosine, 6. Guanosine, 7. Tryptophan and 8. Adenosine.

**Figure 5:**
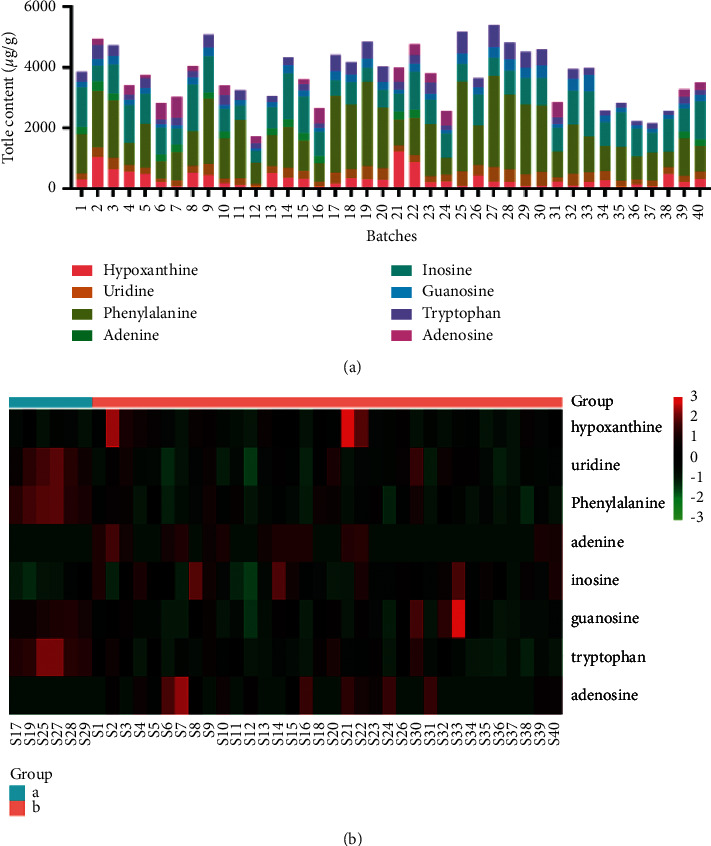
Total content of 8 targets ingredients in 40 batches *Pheretima* (a). The heatmap for the contents of *Pheretima* (“a” representedstronger activity group, “b” represented a lower activity group. The color of the bar represents relative content) (b).

Comparing with the reference standards, eight peaks were identified as hypoxanthine, uridine, phenylalanine, adenine, inosine, guanosine, tryptophan, and adenosine (Figure 4 and Figure S1). The optimized ultrasonic-extraction procedure and HPLC-DAD method were triplicately performed for extracting and determining eight targets content in 40 batches of *Pheretima*. The concentrations of adenine and adenosine did not reach LOQ in some batches (Figure 5(a) and Table S3). It made sense that adenine and adenosine could not be recognized in common peaks of the chemical fingerprint of *Pheretima*. It was obvious that phenylalanine was the highest-content chemical component in most of the samples. The content of phenylalanine ranged from 508.42 ± 20.51 to 2995.55 ± 51.71 *μ*g/g. Thus, phenylalanine might play an important role in *Pheretima* quality control.

### 3.7. Comparison with Other Methods

The difference between the established and reported methods is shown in Table 4. Multiple kinds of chemical components, including anomic acid, nucleoside, and nucleobase, were simultaneously detected in one run. What's more, the derivative reagents were not used, which simplified the operating steps. The established HPLC-DAD method was an alternative method for establishing fingerprint and quantification of efficiency Q-markers of *Pheretima*.

### 3.8. Anticoagulation Activity

Thrombin and fibrinogen played key roles in the coagulation cascade. The former could induce platelet aggregation and fibrin formation [53]. However, the therapeutic basis of the *Pheretima* anticoagulation effect was ambiguous. The ability to inhibit thrombin-induced fibrinogen activation was determined. Quality control (QC) sample replacing the extract with water was used to avoid false positive results. After adding three drops of thrombin, fibrin appears in QC. Therefore, the sample was considered to have no anticoagulation effect if the number of drops was no more than three. Finally, the anticoagulation potency unit of all of the samples was determined (Table S3). The anticoagulation activity of *Pheretima* ranged from 0 to 20800 U/g. The anticoagulation activity of each sample was greatly various. It indicated that the anticoagulation activity-based quality control method of *Pheretima* was urgent to be established.

### 3.9. Screen Q-Markers with Anticoagulation by Grey Relational Analysis

Considering 6 components recognized in the HPLC fingerprint chromatogram, grey relational analysis was used to clarify the relation between component contents and total anticoagulation activity. The results showed that the correlations of hypoxanthine, uridine, phenylalanine, inosine, guanosine, and tryptophan are 0.87, 0.90, 0.71, 0.72, 0.92, and 0.91, respectively. It indicated that the contents of uridine, guanosine, and tryptophan possess a high correlation with the anticoagulation effect. According to anticoagulation activities, samples were divided into two groups by cluster analysis. The quantitative matrix was subjected to WeiShengxin (http://www.bioinformatics.com.cn/) to afford heatmap for adding direct comparison. As shown in Figure 5(b), comparable distribution patterns appeared between “A” and “B” major appearances were found in four components, namely uridine, phenylalanine, guanosine, and tryptophan, which indicated that they had a positive correlation between content and activity.

In order to explore the anticoagulation contribution of these screened six compounds, the mixture reference standard (10-fold concentration of sample S27) was used to detect anticoagulation activity. It was found that the mixture reference standards solution showed no anticoagulation activity and no contribution to the total anticoagulation activity of *Pheretima. S*ome compounds with direct inhibitory fibrinogen activity in *Pheretima* extract solution needed to be further clarified. It was reported that lumbrokinase was the representative and mainly anticoagulation enzyme. The contents of lumbrokinase in *Pheretima* could be determined by a thrombin-induced fibrinogen activation assay, which is similar to the method used in this paper [54, 55]. Based on the facts, there were no quality markers that were related to efficacy. The results of grey relational analysis and heatmap indicate that these six compounds had a positive relationship with the anticoagulation activity of *Pheretima.* Thus, these six compounds were selected as indirect markers of anticoagulation activity.

## 4. Conclusion

In the present work, an efficient and simple method, chemical fingerprint coupled with network pharmacology, has been established for screening potential efficacy-related quality markers of *Pheretima*. Six ingredients (hypoxanthine, uridine, phenylalanine, inosine, guanosine, and tryptophan) were highlighted as *Pheretima* efficacy-related Q-marker owing to the high positive correlation with anticoagulation activity. Furthermore, it could be concluded that *Pheretima* works against stroke presumably by regulating various targets and pathways, which mainly involved anti-inflammatory and antioxidation. To sum up, the integrated strategy of chemical fingerprint and network pharmacology was meaningful to discover the efficacy related Q-markers and improve the quality control of *Pheretima*.

## Figures and Tables

**Table 1:** Optimization of the results of the orthogonal experimental design.

No.	Ratio of sample to liquid (A, mg/mL)	Extraction time (B, min)	Extraction temperature (C, °C)	Extraction power (D, W)	Total content (mg/g)
1	10	40	40	220	3.6466
2	10	50	50	290	3.6795
3	10	60	60	360	3.7052
4	20	40	50	360	3.1518
5	20	50	60	220	3.1720
6	20	60	40	290	3.1203
7	30	40	60	290	3.0463
8	30	50	40	360	2.8356
9	30	60	50	220	3.0658
K1	3.677	3.282	3.201	3.295	
K2	3.148	2.229	3.299	3.282	
K3	2.983	3.297	3.308	3.231	
R	0.694	0.068	0.107	0.064	

Order	*A* > *C* > *B* > *D*				
Optimal level	10	60	60	220	
Optimal combination	10 mg/mL, 60 min, 60°C, 220 W				

Note: Ki: the average of each level in each factor; R: the difference between the maximum and minimum levels of each factor.

**Table 2:** Standard curve, correlation coefficient, linear range, LOD, and LOQ of eight analytes.

Compounds	Regression equation	*r * ^2^	Linearity ranges (*μ*g/mL)	LOD (*μ*g/mL)	LOQ (*μ*g/mL)
Hypoxanthine	*Y* = 1.0873X-0.0564	0.9999	0.32–80	0.04	0.13
Uridine	*Y* = 0.4138X-0.1178	0.9996	1.2–60	0.19	0.50
Phenylalanine	*Y* = 0.5921X-0.3439	0.9998	1.2–300	0.24	0.79
Adenine	*Y* = 1.4172X-1.4904	0.9995	1.5–100	0.44	1.21
Inosine	*Y* = 1.2245X+0.0113	0.9999	0.6–150	0.17	0.58
Guanosine	*Y* = 0.5712X-0.0411	0.9996	1.2–60	0.42	0.96
Tryptophan	*Y* = 1.714X-0.8047	0.9998	2.8–140	0.32	1.14
Adenosine	*Y* = 1.0204X-0.3332	0.9998	1.28–160	0.35	0.97

**Table 3:** The results of precision, stability, and repeatability (*n* = 6).

Compounds	Precision RSD (%)	Stability RSD (%)	Repeatability RSD (%)	Accuracy	
				Recovery (%)	RSD (%)
Hypoxanthine	1.49	3.46	3.71	98.1	1.97
Uridine	1.35	1.70	2.98	102	2.29
Phenylalanine	0.52	1.38	1.21	99.2	1.51
Adenine	1.59	1.64	3.07	101	1.98
Inosine	0.36	2.26	1.29	98.4	2.06
Guanosine	2.65	2.33	2.54	102	2.15
Tryptophan	0.82	2.37	2.58	101	3.28
Adenosine	2.85	4.38	4.95	102	2.12

**Table 4:** Comparison of the LC method with other methods.

Sample amounts (g)	Analytes	Type of solvent	Solvent volume (mL)	Extract method	Derivative reagent	Detection method	Detection time (min)	Reference
10	Aspartic acid, glutamate, serine, glycine, histidine, arginine, threonine, alanine, proline, cysteine, valine, methionine, isoleucine, isoleucine, tyrosine, phenylalanine, lysine	Water	100	Soak for 60 min and ultrasonic bath at room temperature for 60 min	Phenyl isothiocyanate, triethylamine	HPLC	60	[48]
1	Glycine, alanine, valine, leucine, lysine	Water	10	Soak for 12 h and ultrasonic bath at room temperature for 60 min	2,4-Dinitrofluorobenzene	HPLC	72	[49]
1	Hypoxanthine, xanthine, uridine, inosine, guanosine, adenosine	5% Methanol	20	Ultrasonic bath at room temperature for 40 min	—	UPLC	15	[50]
0.5	Uracil, hypoxanthine, xanthine, guanosine, inosine, uridine, 2′-deoxyinosine	Water	25	Ultrasonic bath at room temperature for 30 min	—	HPLC	36	[51]
1	Uracil, hypoxanthine, xanthine, adenosine, inosine	Normal saline	20	Soak for 20 min and ultrasonic bath at room temperature for 40 min	—	HPLC	40	[52]
0.1	Hypoxanthine, uridine, phenylalanine, adenine, inosine, guanosine, tyrosine, adenosine	Water	10	Soak for 10 min and ultrasonic bath at 40 °Cfor 40 min	—	HPLC	60	This work

## Data Availability

All the datasets presented in this study are included in the article.

## Supplementary Materials

Supplementary Materials Figure S1. The extract DAD spectra of eight compounds in mixed reference standards (A) and *Pheretima* (B). Table S1. The similarity indexes between each sample and control chromatogram. Table S2. The annotation of GO enrichment. Table S3. The results of quantification (*μ*g/g, *n* = 3) and anticoagulation activity (U/g) of *Pheretima*.
