# Supplementary material for: An Integrated Strategy of Chemical Fingerprint and Network Pharmacology for the Discovery of Efficacy-Related Q-Markers of Pheretima
Source: Int J Anal Chem. 2022 Oct 4;2022:8774913. doi: 10.1155/2022/8774913 (PMC9553678; doi:10.1155/2022/8774913)
Supplement: Supplementary Materials — Figure S1. The extract DAD spectra of eight compounds in mixed reference standards (A) and Pheretima (B). Table S1. The similarity indexes between each sample and control chromatogram. Table S2. The annotation of GO enrichment. Table S3. The results of quantification (μg/g, n = 3) and anticoagulation activity (U/g) of Pheretima. [file 8774913.f1.docx]

**An integrated strategy of chemical fingerprint and network pharmacology for the discovery of efficacy** **related Q-markers of *Pheretima***

**Ye Shang^1,2^, Suyi Liu****^1,2^, Chunxiao Liang^1,2^, Kunze Du^1,2^, Shujing Chen^1,2^, Jin Li^1^, Hua Jin^1,3^*, Yanxu Chang^1,2^***

^1^ State Key Laboratory of Component-based Chinese Medicine, Tianjin University of Traditional Chinese Medicine, Tianjin, 301617, China

^2^Tianjin Key Laboratory of Phytochemistry and Pharmaceutical Analysis, Tianjin University of Traditional Chinese Medicine, Tianjin, 301617, China

^3^College of Traditional Chinese Medicine, Tianjin University of Traditional Chinese Medicine, Tianjin 300193, China

*Corresponding author:

Yan-xu Chang, Tianjin State Key Laboratory of Modern Chinese Medicine, Tianjin University of Traditional Chinese Medicine

E-mail: Tcmcyx@tjutcm.edu.cn (Y. Chang) and Jinhua77@tjutcm.edu.cn (H.Jin)


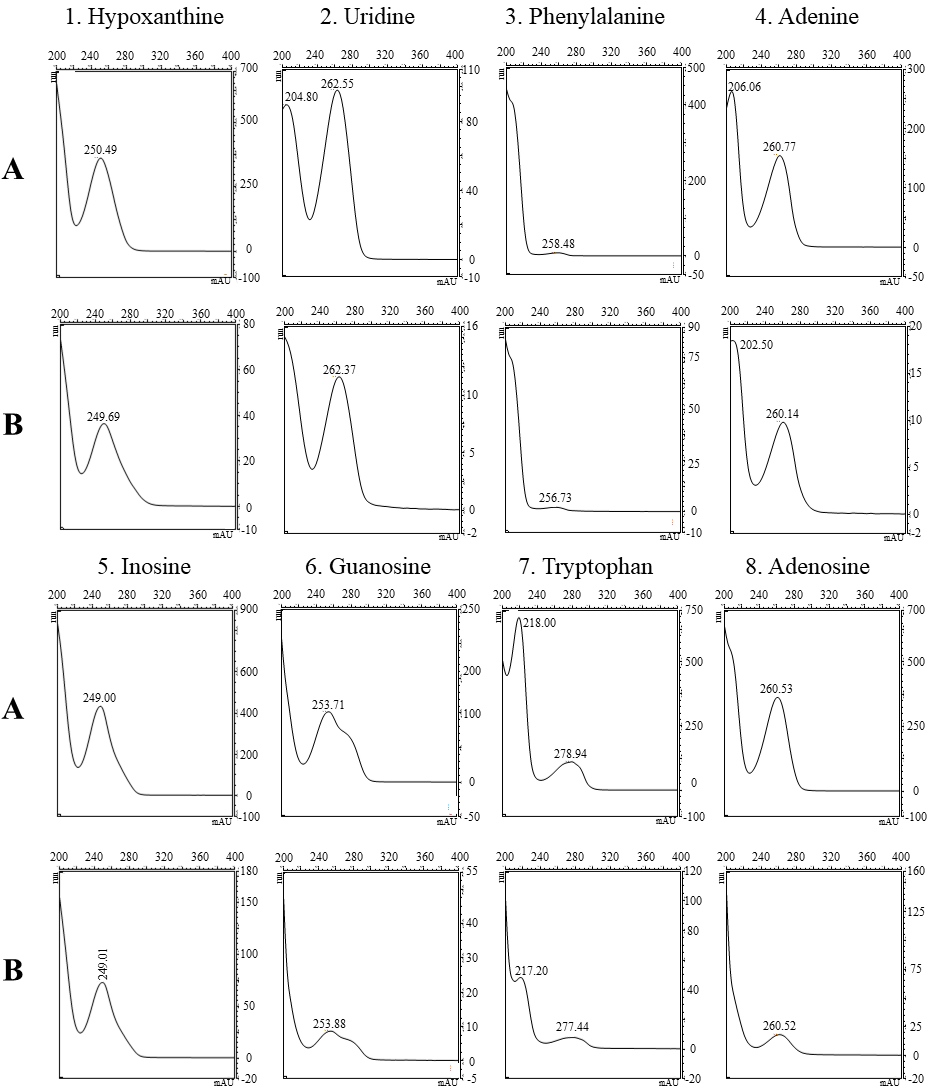


**Figure S1.** The extracted DAD spectra of eight compounds in mixed standards **(A)** and *Pheretima* **(B)**.

**Table S1.** The similarity indexes between each sample and control chromatogram.

| No. | Similarity | No. | Similarity | No. | Similarity | No. | Similarity |
| --- | --- | --- | --- | --- | --- | --- | --- |
| S1 | 0.978 | S11 | 0.921 | S21 | 0.729 | S31 | 0.978 |
| S2 | 0.847 | S12 | 0.988 | S22 | 0.942 | S32 | 0.984 |
| S3 | 0.986 | S13 | 0.966 | S23 | 0.985 | S33 | 0.941 |
| S4 | 0.904 | S14 | 0.96 | S24 | 0.936 | S34 | 0.964 |
| S5 | 0.994 | S15 | 0.956 | S25 | 0.849 | S35 | 0.933 |
| S6 | 0.921 | S16 | 0.926 | S26 | 0.992 | S36 | 0.935 |
| S7 | 0.987 | S17 | 0.882 | S27 | 0.87 | S37 | 0.987 |
| S8 | 0.938 | S18 | 0.964 | S28 | 0.939 | S38 | 0.877 |
| S9 | 0.999 | S19 | 0.856 | S29 | 0.947 | S39 | 0.996 |
| S10 | 0.995 | S20 | 0.926 | S30 | 0.959 | S40 | 0.927 |

**Table S2.** The annotation of GO enrichment

| GO ID | Term | GO ID | Term |
| --- | --- | --- | --- |
| GO:0042493 | response to drug | GO:0045121 | membrane raft |
| GO:0071222 | cellular response to lipopolysaccharide | GO:0072562 | blood microparticle |
| GO:0018105 | peptidyl-serine phosphorylation | GO:0005886 | plasma membrane |
| GO:0043066 | negative regulation of apoptotic process | GO:0005788 | endoplasmic reticulum lumen |
| GO:0006508 | proteolysis | GO:0005768 | endosome |
| GO:0010628 | positive regulation of gene expression | GO:0019899 | enzyme binding |
| GO:0046777 | protein autophosphorylation | GO:0004252 | serine-type endopeptidase activity |
| GO:0008284 | positive regulation of cell proliferation | GO:0042802 | identical protein binding |
| GO:0007165 | signal transduction | GO:0030235 | nitric-oxide synthase regulator activity |
| GO:0008285 | negative regulation of cell proliferation | GO:0005102 | receptor binding |
| GO:0005615 | extracellular space | GO:0008236 | serine-type peptidase activity |
| GO:0005576 | extracellular region | GO:0005524 | ATP binding |
| GO:0070062 | extracellular exosome | GO:0008201 | heparin binding |
| GO:0005829 | cytosol |  |  |

**Table S3.** The results of quantification (μg/g, *n*=3) and anti-coagulation activity (U/g) of *Pheretima*.

| No. | Hypoxanthine | Uridine | Phenylalanine | Adenine | Inosine | Guanosine | Tryptophan | Adenosine | Potency unit |
| --- | --- | --- | --- | --- | --- | --- | --- | --- | --- |
| S1 | 305.17±6.71 | 194.23±6.02 | 1307.18±20.25 | 237.34±1.33 | 1291.95±15.75 | 209.01±4.94 | 311.51±3.64 | a | b |
| S2 | 1046.27±16.49 | 315.91±6.80 | 1877.63±28.39 | 308.90±0.78 | 506.81±18.95 | 219.16±4.72 | 462.42±9.82 | 212.04±8.52 | 50 |
| S3 | 646.92±17.48 | 378.45±13.69 | 1895.33±30.62 | 217.56±5.58 | 968.41±8.45 | 278.60±6.48 | 345.70±11.26 | a | 50 |
| S4 | 563.61±21.23 | 219.02±8.32 | 729.09±20.24 | a | 1259.53±23.28 | 171.81±0.62 | 149.78±4.69 | 317.1±7.76 | b |
| S5 | 487.10±3.92 | 214.19±1.88 | 1455.99±22.90 | a | 969.09±20.42 | 175.48±3.57 | 345.23±10.08 | 103.13±3.12 | 50 |
| S6 | 212.57±4.87 | 132.39±3.23 | 565.53±11.65 | 215.06±5.37 | 891.69±2.90 | 117.83±2.99 | 149.74±2.13 | 536.54±20.41 | 40 |
| S7 | 92.40±0.93 | 178.19±3.28 | 940.13±20.66 | 248.07±0.79 | 524.91±12.96 | 114.79±2.70 | 249.49±3.5 | 676.71±22.91 | 300 |
| S8 | 515.49±23.78 | 240.77±3.69 | 1144.72±31.71 | a | 1538.26±21.98 | 231.87±10.22 | 212.36±1.38 | 163.78±3.90 | 35 |
| S9 | 457.95±5.31 | 359.10±6.71 | 2162.30±62.92 | 191.02±3.38 | 1207.38±39.69 | 288.09±3.72 | 419.82±5.53 | a | 60 |
| S10 | 189.34±7.83 | 156.98±3.99 | 1310.36±30.53 | 228.35±2.29 | 754.70±9.82 | 135.03±5.99 | 302.51±4.32 | 323.68±7.28 | 50 |
| S11 | 142.50±2.37 | 216.67±8.94 | 1907.14±29.29 | a | 498.39±7.60 | 165.91±2.18 | 320.13±10.34 | a | 2800 |
| S12 | 56.64±2.53 | 109.05±1.59 | 693.68±22.75 | a | 390.39±15.31 | 75.38±1.77 | 175.00±2.26 | 226.26±4.08 | b |
| S13 | 508.98±16.84 | 231.22±5.95 | 1037.17±33.67 | 209.92±0.73 | 713.56±11.20 | 152.88±4.33 | 188.27±4.43 | a | 560 |
| S14 | 373.46±2.13 | 325.71±9.88 | 1345.04±5.12 | 242.28±3.98 | 1523.60±29.03 | 267.73±1.46 | 261.77±6.40 | a | 50 |
| S15 | 331.18±12.76 | 262.75±9.38 | 997.14±31.25 | 240.57±4.27 | 1195.58±8.23 | 219.19±5.94 | 204.62±2.93 | 157.47±5.04 | 70 |
| S16 | 75.83±2.03 | 148.49±4.50 | 611.19±19.70 | 235.31±1.47 | 814.04±36.95 | 119.57±2.46 | 163.05±4.82 | 503.28±4.96 | 50 |
| S17 | 174.56±5.80 | 352.70±4.56 | 2523.80±91.95 | a | 528.44±2.40 | 305.29±11.80 | 533.06±12.35 | a | 12000 |
| S18 | 359.47±15.49 | 297.83±3.64 | 2128.21±100.31 | a | 739.40±15.64 | 242.71±5.52 | 400.23±15.23 | a | 2100 |
| S19 | 310.83±9.36 | 438.92±19.73 | 2786.13±58.00 | a | 453.49±11.05 | 299.56±9.46 | 556.24±12.47 | a | 12800 |
| S20 | 291.90±8.36 | 398.02±15.00 | 2002.65±37.31 | a | 570.98±11.00 | 266.77±8.37 | 500.53±20.02 | a | 2800 |
| S21 | 1236.46±47.91 | 188.78±2.83 | 859.31±27.53 | 254.25±1.95 | 592.57±25.71 | 137.31±2.34 | 267.60±7.29 | 461.08±4.64 | 300 |
| S22 | 876.71±26.97 | 228.82±4.49 | 1240.33±53.86 | 260.20±2.48 | 1256.77±31.33 | 267.55±6.31 | 283.27±1.46 | 360.4±6.45 | 45 |
| S23 | 203.52±6.06 | 224.84±4.02 | 1719.91±36.94 | a | 795.20±37.57 | 183.16±6.33 | 379.08±6.31 | 304.54±5.17 | 1000 |
| S24 | 234.79±7.56 | 235.31±6.21 | 559.70±14.33 | a | 794.74±27.03 | 108.17±0.68 | 154.42±3.17 | 479.68±19.87 | 50 |
| S25 | 102.12±0.94 | 475.62±4.98 | 2965.34±134.48 | a | 584.16±18.87 | 342.59±10.23 | 707.01±22.08 | a | 20800 |
| S26 | 446.09±13.42 | 336.52±13.14 | 1317.14±48.23 | a | 1003.65±22.84 | 227.66±7.37 | 319.29±4.75 | a | 200 |
| S27 | 221.55±4.65 | 501.70±18.99 | 2995.55±51.71 | a | 604.47±9.37 | 356.24±10.76 | 707.99±11.2 | a | 16000 |
| S28 | 216.74±8.51 | 432.04±19.46 | 2450.70±106.18 | a | 801.97±39.37 | 362.51±15.09 | 558.68±13.13 | a | 16000 |
| S29 | 101.10±2.16 | 376.62±15.11 | 2310.39±30.19 | a | 870.47±32.38 | 331.52±12.29 | 530.43±17.49 | a | 8800 |
| S30 | 108.21±4.31 | 450.70±7.85 | 2203.16±63.42 | a | 896.76±16.71 | 412.62±12.05 | 527.79±13.39 | a | 4800 |
| S31 | 227.79±2.35 | 160.48±0.98 | 841.95±10.47 | a | 784.68±32.80 | 129.89±2.36 | 219.80±4.71 | 486.39±7.68 | b |
| S32 | 119.49±3.73 | 373.22±14.96 | 1641.88±71.47 | a | 1109.88±50.91 | 384.05±13.69 | 316.65±7.18 | a | 250 |
| S33 | 219.31±2.25 | 323.56±14.65 | 1177.88±41.82 | a | 1495.54±32.94 | 539.91±21.53 | 226.02±5.93 | a | 60 |
| S34 | 275.62±9.78 | 309.64±4.82 | 816.04±14.89 | a | 803.25±12.33 | 229.21±10.77 | 146.09±0.72 | a | 50 |
| S35 | 59.07±1.75 | 210.62±4.12 | 1107.46±24.62 | a | 1134.33±19.48 | 173.86±8.27 | 134.74±4.00 | a | 25 |
| S36 | 159.65±2.85 | 153.71±2.00 | 747.27±6.53 | a | 917.22±6.51 | 123.76±4.73 | 133.01±1.49 | a | 25 |
| S37 | 88.48±3.47 | 182.34±6.74 | 926.28±18.05 | a | 645.34±28.58 | 136.68±1.20 | 191.24±3.00 | a | 60 |
| S38 | 484.52±22.37 | 235.02±8.03 | 508.42±20.51 | a | 1067.41±37.77 | 157.37±7.27 | 115.55±0.71 | a | 100 |
| S39 | 214.28±6.75 | 224.18±8.29 | 1220.55±53.11 | 230.86±2.59 | 755.96±8.76 | 157.08±7.37 | 217.63±6.91 | 250.95±5.03 | 55 |
| S40 | 326.05±9.91 | 243.34±5.83 | 836.62±13.56 | 218.38±4.60 | 1264.65±16.81 | 195.98±4.92 | 171.14±1.59 | 256.84±10.01 | 40 |

Note: a: concentration less than LOQ; b: potency unit less than QC.
